# Targeting IRE1 endoribonuclease activity alleviates cardiovascular lesions in a murine model of Kawasaki disease vasculitis

**DOI:** 10.1172/jci.insight.157203

**Published:** 2022-03-22

**Authors:** Stefanie Marek-Iannucci, Asli D. Yildirim, Syed M. Hamid, Asli B. Ozdemir, Angela C. Gomez, Begüm Kocatürk, Rebecca A. Porritt, Michael C. Fishbein, Takao Iwawaki, Magali Noval Rivas, Ebru Erbay, Moshe Arditi

**Affiliations:** 1 Graduate School of Biomedical Sciences,; 2 Department of Pediatrics, Division of Infectious Diseases and Immunology,; 3 Infectious and Immunologic Diseases Research Center and Department of Biomedical Sciences, and; 4 Smidt Heart Institute, Cedars-Sinai Medical Center, Los Angeles, California, USA.; 5 Department of Pathology, David Geffen School of Medicine at UCLA, Los Angeles, California, USA.; 6 Department of Life Science, Medical Research Institute, Kanazawa Medical University, Ishikawa, Japan.

**Keywords:** Immunology, Vascular Biology, Cell stress, Mouse models, Vasculitis

## Abstract

Kawasaki disease (KD) is the leading cause of noncongenital heart disease in children. Studies in mice and humans propound the NLRP3/IL-1β pathway as the principal driver of KD pathophysiology. Endoplasmic reticulum (ER) stress can activate the NLRP3 inflammasome, but the potential implication of ER stress in KD pathophysiology has not been investigated to our knowledge. We used human patient data and the *Lactobacillus casei* cell wall extract (LCWE) murine model of KD vasculitis to characterize the impact of ER stress on the development of cardiovascular lesions. KD patient transcriptomics and single-cell RNA sequencing of the abdominal aorta from LCWE-injected mice revealed changes in the expression of ER stress genes. Alleviating ER stress genetically, by conditional deletion of inositol-requiring enzyme 1 (IRE1) in myeloid cells, or pharmacologically, by inhibition of IRE1 endoribonuclease (RNase) activity, led to significant reduction of LCWE-induced cardiovascular lesion formation as well as reduced caspase-1 activity and IL-1β secretion. These results demonstrate the causal relationship of ER stress to KD pathogenesis and highlight IRE1 RNase activity as a potential new therapeutic target.

## Graphical abstract

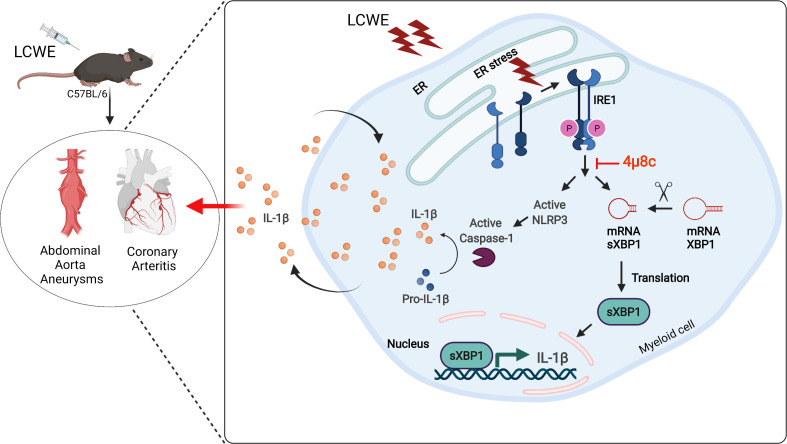

## Introduction

Kawasaki disease (KD), a febrile systemic vasculitis of unknown etiology, predominantly affects children under 5 years of age (1–3). Although the acute symptoms of KD are self-limiting, inadequate treatment can result in the formation of long-term cardiovascular sequelae, such as coronary artery aneurysms (CAAs), which correlate with an elevated risk for myocardial infarction, sudden cardiac death, and heart failure in early adulthood (4). Approximately 5% of all acute coronary syndromes in young adults can be attributed to CAA due to KD in childhood, highlighting the importance of developing efficient therapies for KD and reducing the likelihood of long-term complications (3). The current gold standard treatment, high-dose intravenous immunoglobulin (IVIG), can diminish CAA occurrence, yet about one-fifth of patients are unresponsive to IVIG and require additional interventions (3, 5).

The *Lactobacillus casei* cell wall extract (LCWE) murine model of KD vasculitis mimics histological, functional, and immunological characteristics seen in human KD vasculitis (6, 7). The leucine-rich repeat–containing protein 3 (NLRP3) inflammasome and interleukin-1β (IL-1β) pathway have been shown to be major drivers of cardiovascular lesion formation in this model (8–11). The major sources of IL-1β in the LCWE model are monocytes, macrophages, and dendritic cells that infiltrate the inflamed vascular tissues (10). Vascular smooth muscle cells (VSMCs) surrounding the CAA respond to IL-1β, which triggers a pathogenic reprograming of VSMCs toward the proliferative type 2 phenotype, which further promotes cardiovascular lesions (10). Blocking the IL-1β pathway in mice, genetically or pharmacologically with either neutralizing antibodies or small-molecule inhibitors of NLRP3, prevents the development of LCWE-induced KD vasculitis (8–10). Human patients with KD also exhibit elevated levels of circulating IL-1β and increased expression of IL-1 pathway–associated genes during the early stage of disease (10, 12–15). Further supporting the importance of this pathway, IL-1β polymorphisms are associated with increased IL-1β production and IVIG resistance in patients with KD (14, 16), and anakinra, an IL-1 receptor antagonist, has successfully been used to treat patients with IVIG-resistant KD (17–21).

The endoplasmic reticulum (ER) is responsible for protein synthesis and processing and cellular calcium homeostasis (22). Accumulation of misfolded proteins or disruption of calcium homeostasis triggers ER stress and activates the unfolded protein response (UPR) pathway, which aims to reestablish homeostasis within the ER by easing the burden of protein translation and eliminating misfolded proteins (22). The UPR is composed of 3 mechanistically distinct arms mediated by ER-resident protein folding sensors inositol-requiring enzyme 1 (IRE1), PKR-like endoplasmic reticulum kinase (PERK), and activating transcription factor 6 (ATF6) (22, 23). UPR activation is also a hallmark of low-grade, sustained inflammation underlying several metabolic disorders, including dyslipidemia, obesity, and atherosclerosis (22, 24, 25). Genetic or chemical inhibition of 2 UPR regulators, namely IRE1 and PERK, has been shown to decrease inflammation both in vitro and in vivo (26, 27). IRE1 consists of endonuclease and kinase domains (28). Under conditions of ER stress, IRE1 autophosphorylates, activating its ability to splice the mRNA encoding X-box binding protein 1 (XBP1), an important transcription factor in the UPR pathway (29). IRE1 RNase activity has been shown to induce NLRP3 and IL-1β in macrophages stimulated by saturated fatty acids (27, 30, 31). Similarly, accumulation of ER stress markers in hepatocytes is associated with NLRP3 inflammasome activation in an IRE1-dependent manner (32). Furthermore, under conditions of ER stress, the activation of IRE1 leads to accumulation of mitochondrial reactive oxygen species, which are known to activate NLRP3 (27, 33). In addition to activating the UPR, disruption of calcium homeostasis is known to activate the NLRP3 inflammasome (34).

Given these established connections between ER stress and NLRP3 inflammasome activation, we asked whether ER stress–mediated IRE1 activation plays a role in the development of cardiovascular lesions in KD. Analysis of publicly available gene expression data sets revealed changes in the expression of ER stress–related genes in whole blood of patients with KD. In the murine model of KD vasculitis, we show increased ER stress in cardiovascular lesions upon LCWE injection. We also demonstrate that genetic and pharmacological modulations of the IRE1 pathway mitigate inflammation by hindering NLRP3 activation and IL-1β secretion.

## Results

### Increased expression of ER stress signature genes during human KD.

**Figure 1:**
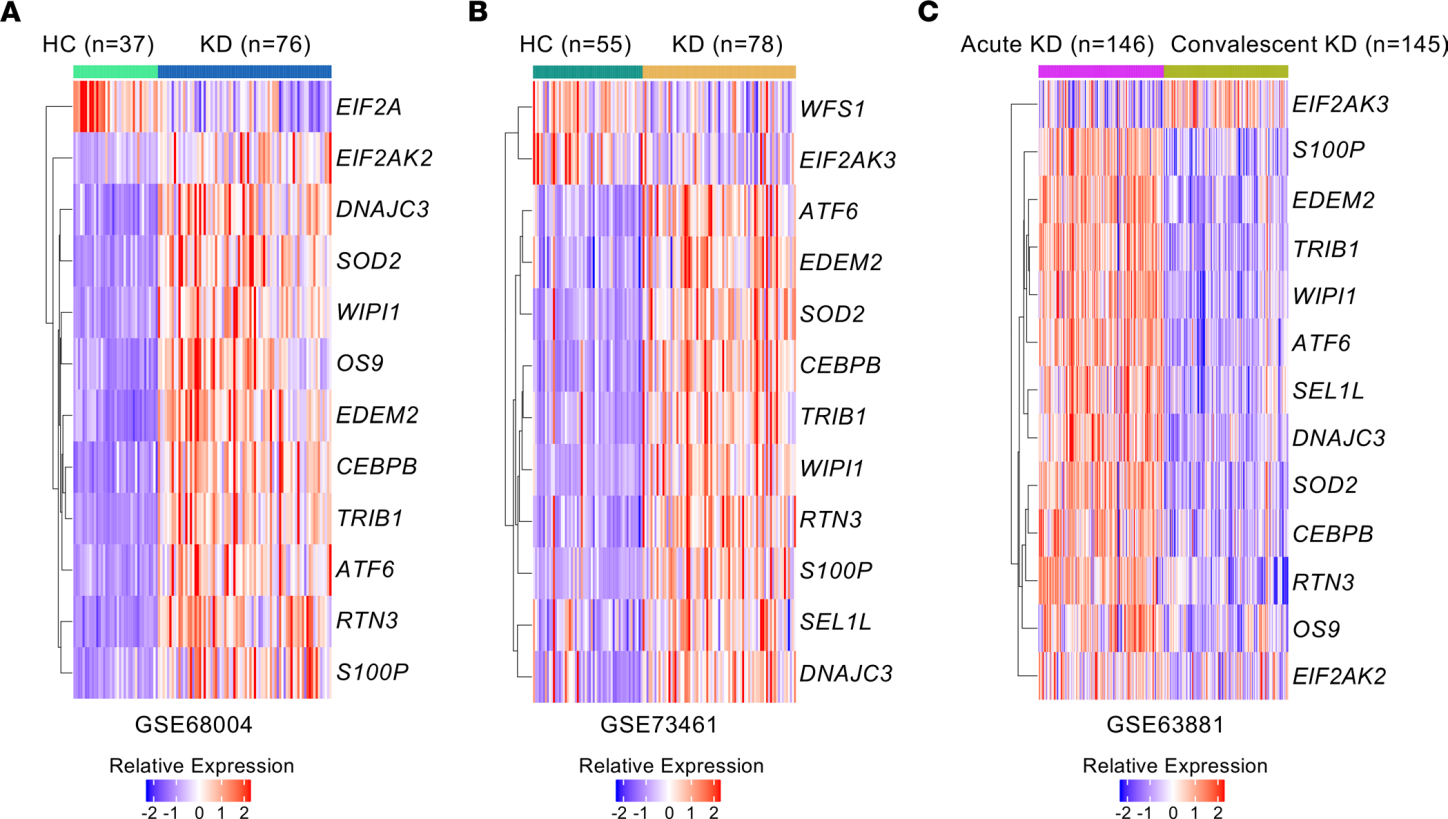
Change in the expression of ER stress signature genes during acute KD. (**A** and **B**) Heatmaps showing ER stress signature differentially expressed genes (DEGs; at least 1.5-fold change [FC] in either direction with an adjusted *P* < 0.05) in whole blood from patients with acute KD compared with HCs (**A** GSE68004; KD patients *n* = 76 and HCs *n* = 37 and **B** GSE73461; KD patients *n* = 78 and HCs *n* = 55). (**C**) Expression of ER stress DEGs identified in **A** and **B** in whole blood of acute KD compared with convalescent IVIG-treated KD patients (GSE63881; acute KD patients *n* = 146 and convalescent KD patients *n* = 145). Genes selected based on adjusted *P* < 0.05. (**A**–**C**) Blue-red color gradient: low to high expression relative to the mean of each row. Each column represents 1 patient of the defined groups. Differential expression was analyzed with GEO2R.

To determine if disruption of ER homeostasis and ER stress are involved in acute KD, we generated an ER stress gene signature composed of 29 genes known to be downstream of the 3 UPR arms IRE1/XBP1, ATF6, and PERK/ATF4 (Table 1). We next examined changes in expression of these signature genes in 2 publicly available human transcriptomics data sets generated from whole blood of patients with acute KD compared with healthy controls (HCs) (Figure 1, A and B) (35, 36). We observed that 18 and 15 ER stress signature genes were differentially expressed genes (DEGs; adjusted *P* < 0.05) in these 2 data sets respectively. Among these sets of DEGs, 11 and 10 genes from the ER stress signature were upregulated in patients with acute KD (FC > 1.5) (Figure 1, A and B). We next assessed the expression of the identified ER stress DEGs in acute KD patients (Figure 1, A and B) in another gene expression data set comparing whole blood of acute KD patients with IVIG-treated convalescent KD patients (Figure 1C) (37). KD resolution in IVIG-treated convalescent KD patients was associated with decreased expression (adjusted *P* < 0.05) of the upregulated ER stress DEGs (Figure 1C). These findings suggest that disruption of ER homeostasis may contribute to the development of KD vasculitis.

### Infiltration of immune cells expressing ER stress signature genes in abdominal aorta tissues of LCWE-injected mice.

**Figure 2:**
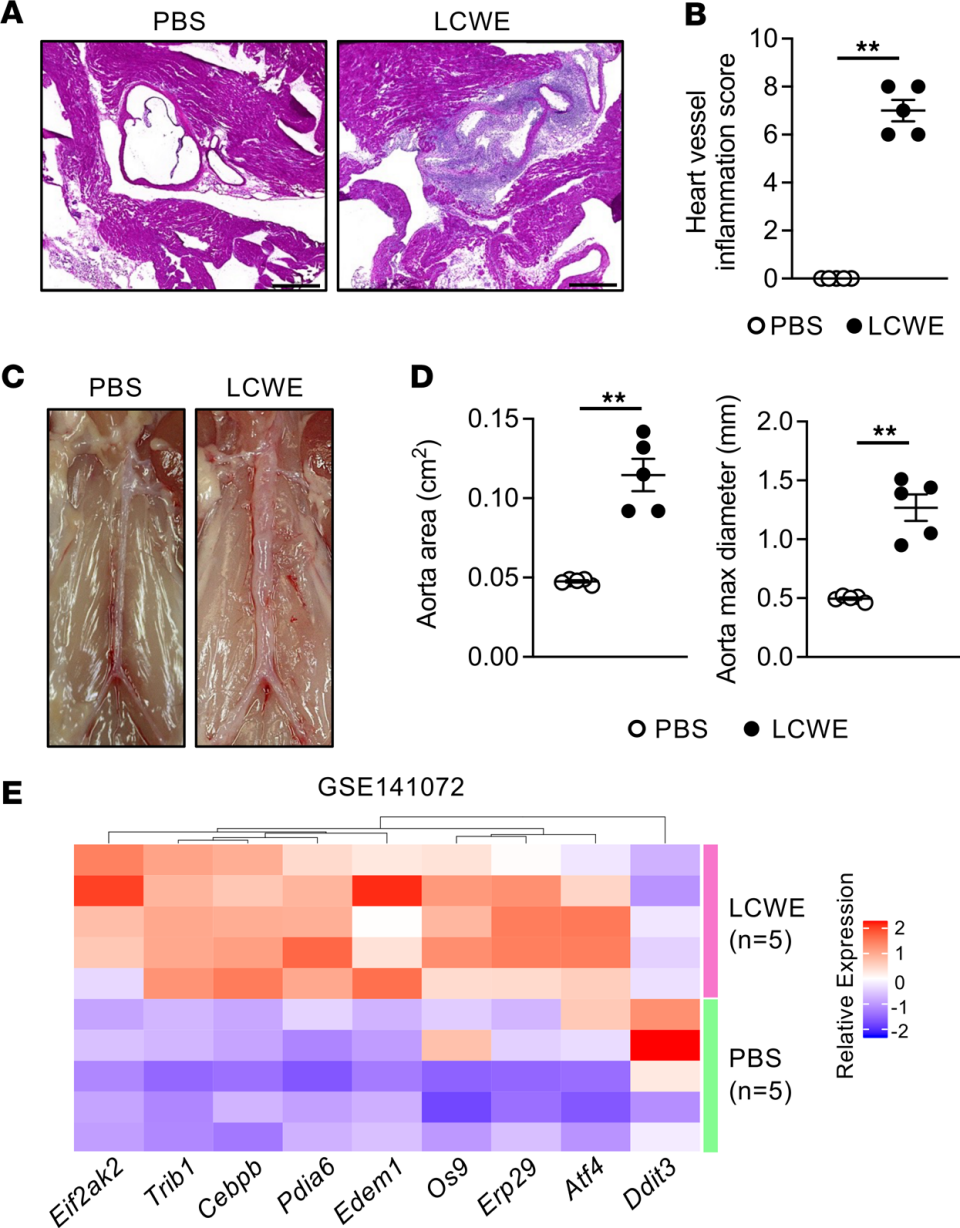
Increased expression of ER stress gene signature in the abdominal aortas of LCWE-injected mice. (**A** and **B**) Representative H&E-stained heart sections (**A**) and heart vessel inflammation scores (**B**) of WT mice injected with either PBS or LCWE at 1 week postinjection (*n* = 5/group). Scale bars: 500 μm. (**C** and **D**) Representative pictures of the abdominal aorta area (**C**), as well as abdominal aorta area and maximal abdominal aorta diameter measurements (**D**) of PBS- and LCWE-injected mice, 1 week after LCWE injection (*n* = 5/group). (**E**) Heatmap illustrating the expression of ER stress signature DEGs (1.5 FC in either direction with an adjusted *P* < 0.05) in abdominal aorta tissues of PBS- and LCWE-injected mice (*n* = 5/groups; GSE141072). Blue-red color gradient: low to high expression relative to the mean of each column. Each row represents 1 mouse of the defined groups. **P < 0.01 by Mann-Whitney test.

We next used the LCWE murine model of KD vasculitis to further characterize the role of ER stress during KD vasculitis. As previously published (8, 9), compared with PBS-injected control mice, LCWE-injected mice developed heart vessel inflammation as well as infrarenal abdominal aorta aneurysms and dilatations (Figure 2, A–D). By analyzing a publicly available bulk RNA sequencing (bulk RNA-Seq) data set generated from abdominal aortas of PBS- and LCWE-injected mice (National Center for Biotechnology Information’s Gene Expression Omnibus GSE141072) (11), we observed that 9 ER stress signature genes were differentially expressed in the abdominal aorta aneurysms of LCWE-injected mice (1.5 FC in either direction and adjusted *P* < 0.05) (Figure 2E). Two of these DEGs (*Cebpb* and *Trib1*) were upregulated not only in abdominal aorta aneurysms of LCWE-injected mice but also consistently in the 3 analyzed gene expression data sets generated from PBMCs of patients with acute KD (Figure 1, A–C). Additionally, 4 of these abdominal aorta aneurysm DEGs (*Eif2ak2*, *Os9*, *Cebpb*, and *Trib1*) were upregulated in PBMCs from patients with acute KD, and their expression was reduced during KD resolution in patients with IVIG-treated convalescent KD (Figure 1C and Figure 2E).

**Figure 3:**
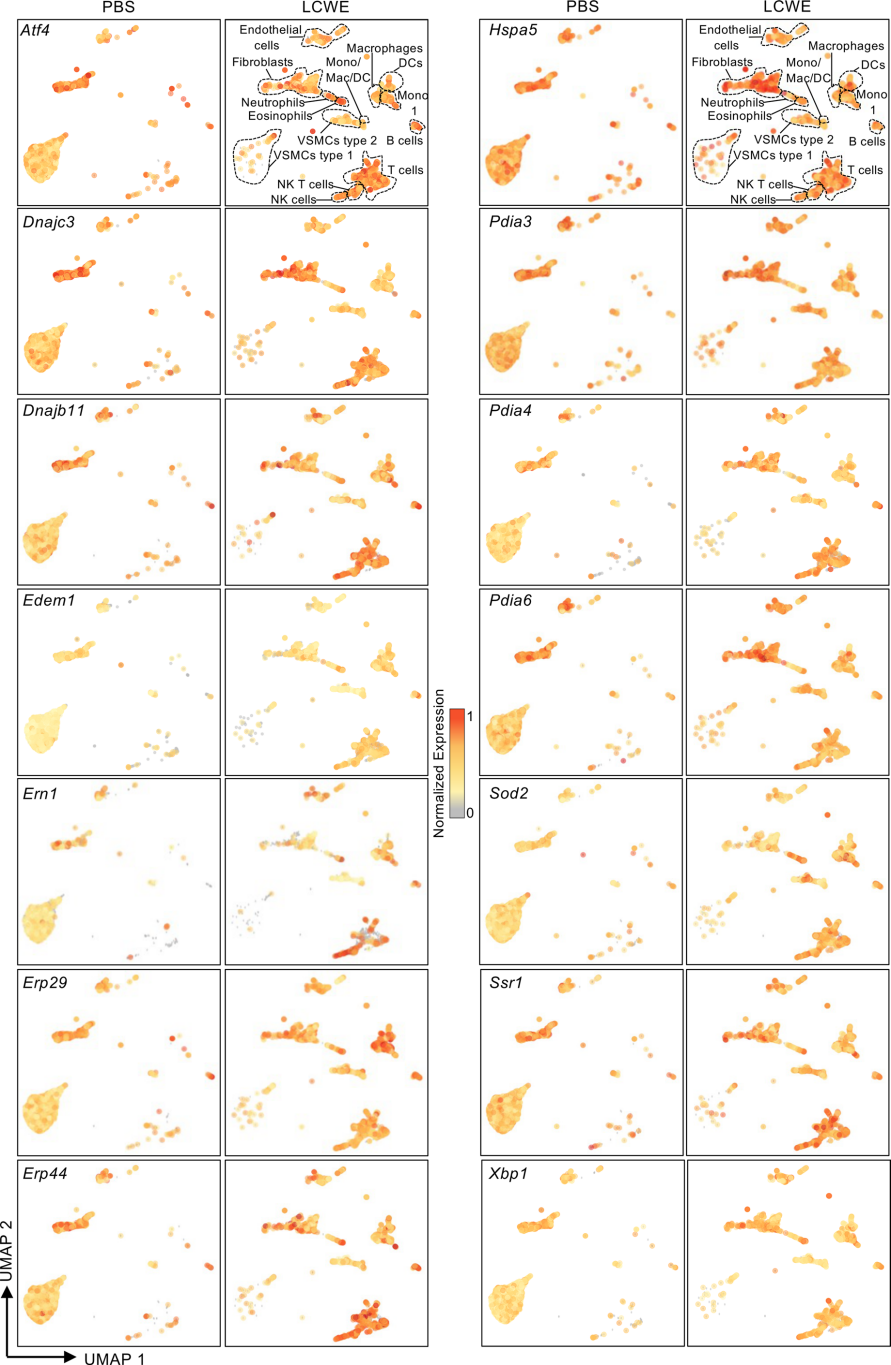
Immune cells infiltrating LCWE-induced abdominal aorta aneurysms express ER stress signature genes. UMAP visualization of a previously published scRNA-Seq data set generated from abdominal aorta tissues of PBS-injected (*n* = 9 tissues pooled) and LCWE-injected (*n* = 6 tissues pooled) mice (National Center for Biotechnology Information’s Gene Expression Omnibus GSE178765) representing a gradient expression of selected ER stress signature genes. Gray-yellow-red gradient: min to max normalization of CP10K expression. CP10K indicates counts per 10,000 reads; Mono, monocytes; NK, natural killer, UMAP, uniform manifold approximation and projection.

Next, to determine which cellular subsets express ER stress–related genes, we analyzed a published single-cell RNA sequencing (scRNA-Seq) data set generated from abdominal aortas collected from PBS-injected (pool of *n* = 9) and LCWE-injected (pool of *n* = 7) mice (GSE178765) (10). Using this scRNA-Seq data set, we previously reported intense infiltrations of immune cells in the abdominal aorta of LCWE-injected mice, which was further validated by flow cytometric analysis (10). In control PBS-injected mice, ER stress signature genes were commonly expressed by stromal cells, such as VSMCs and fibroblasts. However, the immune cells infiltrating the abdominal aortas after LCWE injection also expressed ER stress signature genes (Figure 3). *Ern1*, which encodes IRE1, *Xbp1* and specific *Xbp1* target genes (*Erp44*, *Sod2*, and *Ssr1*), as well as common targets of *Xbp1/Atf6* (*Dnajc3*, *Dnajb11*, *Edem1*, *Hspa5*, *Pdia3*, and *Pdia4*) and *Xbp1*/*Atf4* (*Erp29* and *Hspa5*) were detected in fibroblasts as well as monocytes, macrophages, dendritic cells (DCs), and lymphocytes that infiltrated the abdominal aortas of LCWE-injected mice (Figure 3). Overall, these results suggest that ER stress is increased during LCWE-induced KD vasculitis and may contribute to vascular tissue inflammation.

### Increased protein expression of IRE1-regulated genes during LCWE-induced KD vasculitis.

**Figure 4:**
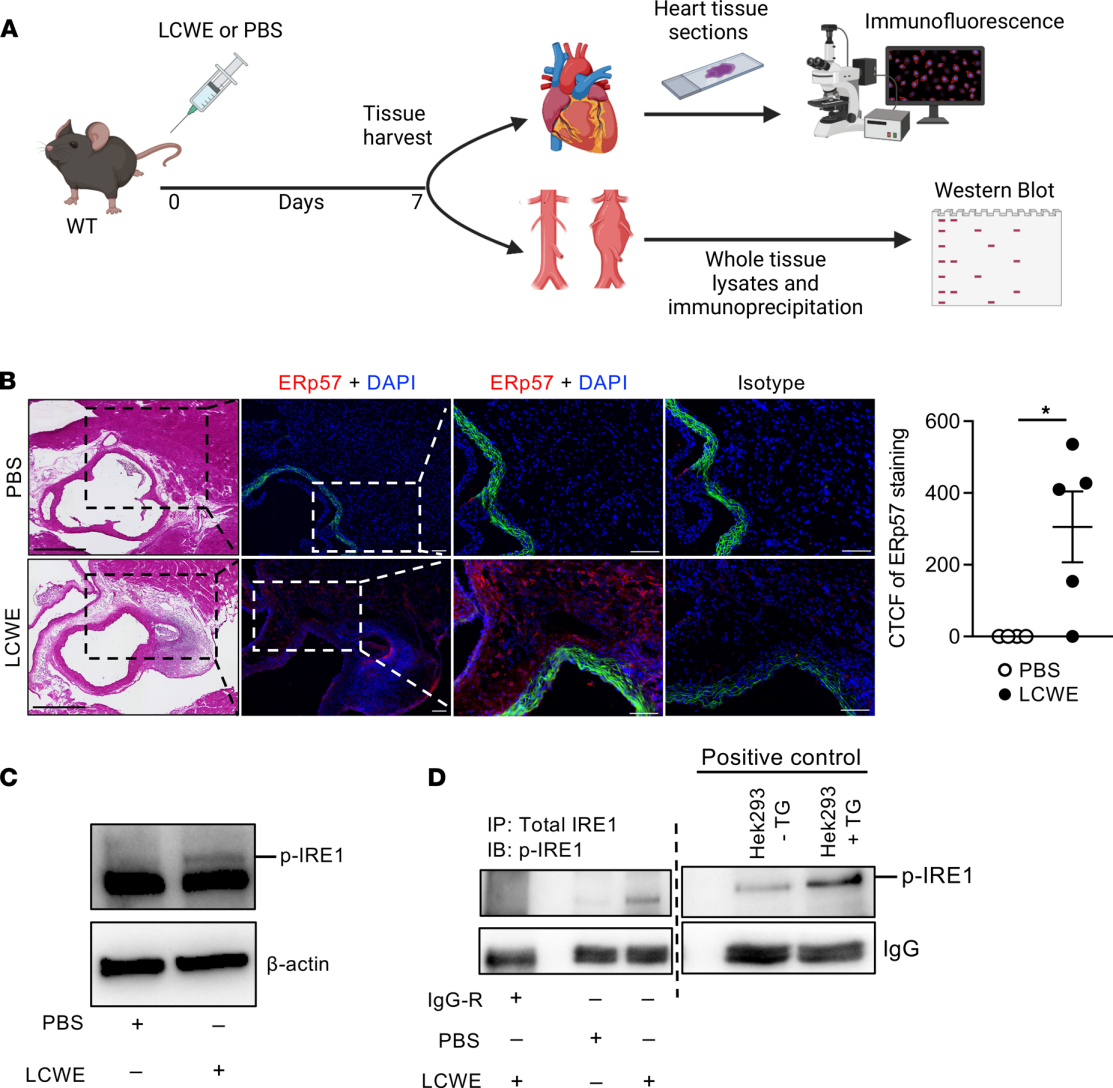
Increased expression of IRE1 pathway–related proteins during LCWE-induced KD vasculitis. (**A**) Experimental schema. WT mice were injected with either PBS or LCWE, and 1 week later, heart tissues and abdominal aortas were collected. Heart tissue sections were used for immunofluorescence staining and abdominal aortas for immunoprecipitation and Western blot analysis. (**B**) H&E and immunofluorescence staining of ERp57 (red) and DAPI (blue) in heart sections of PBS- or LCWE-injected mice, 1 week after injection (*n* = 4–5/group). Scale bars, 100 μm. Quantification of ERp57 fluorescence intensity (right panel). (**C**) Western blot analysis of p-IRE1 (S747) and β-actin in abdominal aorta tissues from PBS-injected (*n* = 3 tissues pooled/lane) and LCWE-injected mice (*n* = 2 tissues pooled/lane) at 1 week postinjection. (**D**) Abdominal aortas were collected from PBS- and LCWE-injected mice, and tissue lysates (*n* = 3/condition) were immunoprecipitated using anti-IRE1 antibody or IgG rabbit as a control. Western blot was performed using anti–phospho-IRE1 (S747) antibody. Lysates from HEK293T cells treated with DMSO and thapsigargine (TG) were also immunoprecipitated with anti-IRE1 and used as positive controls. *P < 0.05 by Mann-Whitney test. CTCF, corrected total cell fluorescence.

We next determined if expression changes in ER stress signature genes observed during LCWE-induced KD vasculitis resulted in altered protein levels of ER stress genes in cardiovascular lesions. WT mice were injected with either PBS or LCWE, and vascular tissues were collected 1 week later (Figure 4A). Compared with PBS-injected control mice, expression of the UPR target protein disulfide-isomerase A3 (PDIA3), also known as ERp57, was higher in heart tissue sections of LCWE-injected mice, indicating increased ER stress (Figure 4B). To evaluate activation of the IRE1 arm of the UPR, we assessed IRE1 phosphorylation by Western blot in the abdominal aortas of PBS- and LCWE-injected mice. Compared with PBS control mice, IRE1 phosphorylation (p-IRE1) was markedly induced in the abdominal aortas of LCWE-injected mice (Figure 4C). Next, we immunoprecipitated total IRE1 from the abdominal aortas of PBS- and LCWE-injected mice and probed with a p-IRE1–specific antibody. Aortas of LCWE-injected mice showed clear induction of p-IRE1 as compared with the PBS control group (Figure 4D). Collectively, these findings indicate that the IRE1 pathway is induced in cardiovascular lesions of LCWE-injected mice.

### Inhibition of IRE1 decreases LCWE-induced inflammasome activation in macrophage cells.

**Figure 5:**
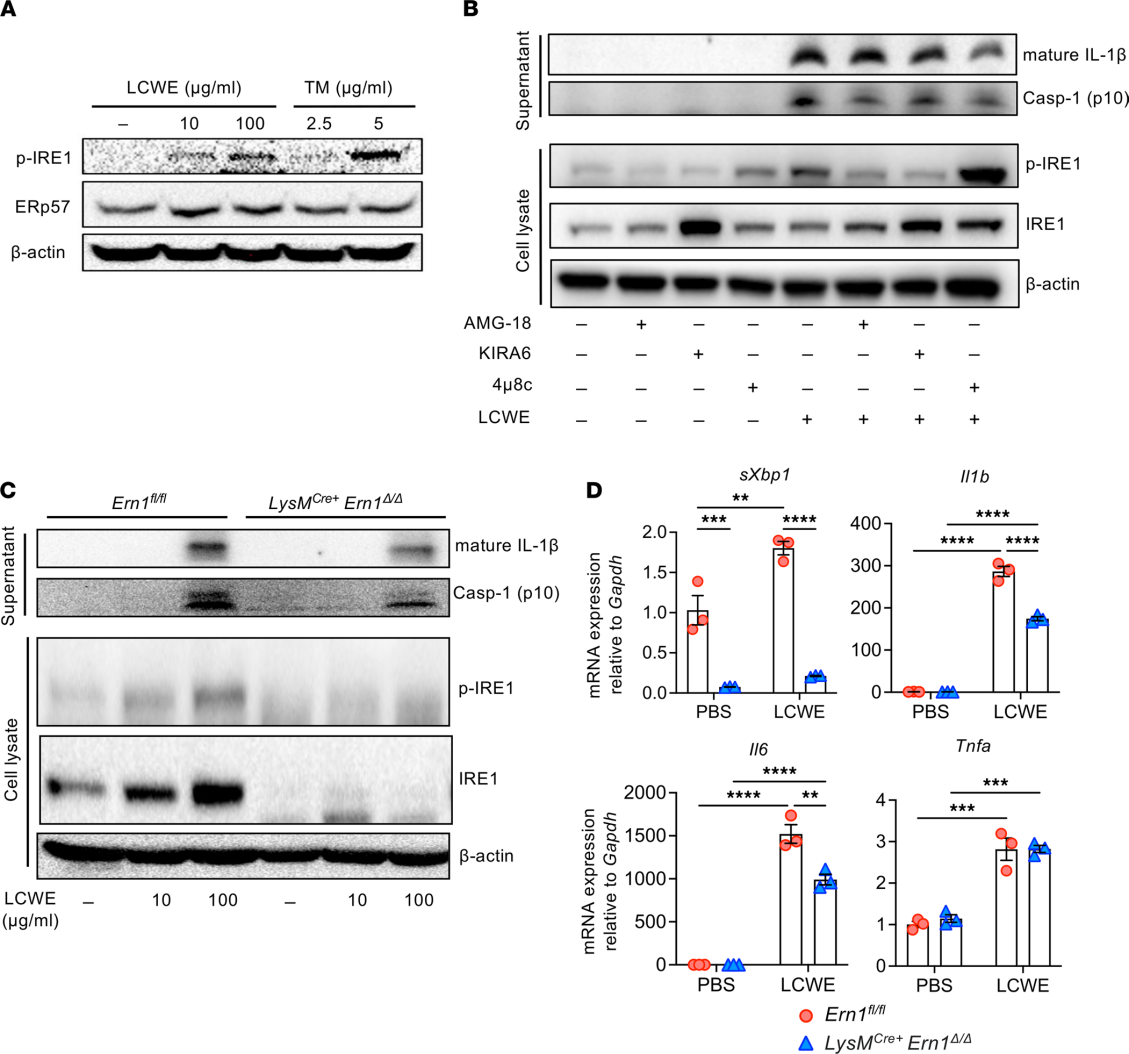
LCWE induces IL-1β and caspase-1 secretion through IRE1. (**A**) WT BMDMs were treated with indicated doses of LCWE for 16 hours. The ER stress inducer tunicamycin (TM) was used as positive control. Protein lysates were analyzed by Western blotting using specific antibodies against p-IRE1 (S747) and ERp57. (**B**) BMDMs were pretreated with IRE1 kinase inhibitors AMG-18 (3 μM) and KIRA6 (15 μM) and RNase inhibitor 4μ8c (100 μM) for 1 hour and then treated with LCWE (75 μg/mL) for 14 hours along with the inhibitors. The supernatants were analyzed by Western blotting for cleaved caspase-1 (p10) and mature IL-1β. Whole-cell lysates were analyzed by Western blotting using specific antibodies against p-IRE1 (S747), IRE1, and β-actin (*n* = 3). (**C**) BMDMs derived from *LysM^Cre+^* *Ern1*^Δ*/*Δ^ and littermate control *Ern1^fl/fl^* mice were treated with indicated doses of LCWE for 16 hours to activate the inflammasome. Supernatants were collected and analyzed by Western blotting for cleaved caspase-1 (p10) and mature IL-1β. Whole-cell lysates were prepared from the cells and analyzed by Western blotting using specific antibodies against IRE1, p-IRE1 (S747), and β-actin (*n* = 3). (**D**) Quantitative real-time PCR (qRT-PCR) of *sXbp1*, *Il1b*, *Il6*, and *Tnfa* transcripts in BMDMs derived from *LysM^Cre+^* *Ern1*^Δ*/*Δ^ and littermate control *Ern1^fl/fl^* mice treated with either PBS or LCWE. **P < 0.01, ***P < 0.001, ****P < 0.0001 by 2-way ANOVA with Tukey’s multiple-comparison test. Casp-1, cleaved caspase-1.

ER stress and IRE1 have previously been linked to NLRP3 inflammasome activation and IL-1β production in various inflammatory conditions (27, 33, 38). However, the contribution of ER stress to NLRP3 activation and IL-1β secretion remains unknown in KD vasculitis. By scRNA-Seq analysis of abdominal aortas and spatial transcriptomics of heart tissues from PBS- and LCWE-injected mice, we have demonstrated that myeloid cells infiltrating the cardiovascular lesions of LCWE-injected mice coexpress *Nlrp3*, *Pycard*, *Casp1*, and *Il1b* and may, therefore, act as source of IL-1β (10). Furthermore, CD11c^+^F4/80^+^ macrophages present in LCWE-induced cardiovascular lesions exhibit a caspase-1 activity (9, 39), and their depletion using clodronate liposomes (clodrosomes) reduces the development of LCWE-induced abdominal aorta aneurysms (9). Since our scRNA-Seq analysis also revealed expression of ER stress–related genes by macrophages and monocytes infiltrating the abdominal aortas of LCWE-injected mice (Figure 3), we assessed the role of ER stress in myeloid cells in response to LCWE. Bone marrow–derived macrophages (BMDMs) were stimulated with either LCWE or the known UPR activator tunicamycin (TM), as a positive control. LCWE stimulation led to a robust induction of p-IRE1 in BMDMs accompanied by increased expression of the chaperone ERp57 (Figure 5A), indicating activation of downstream UPR signaling. We next investigated how inhibition of IRE1’s 2 different enzymatic activities affects the induction of NLRP3 inflammasome by LCWE. LCWE stimulation led to a robust induction of p-IRE1, which was diminished by treatment with the kinase inhibitors AMG-18 and KIRA6, but not by treatment with a specific RNase inhibitor of IRE1, 4μ8c (Figure 5B). Treatment of BMDMs with either the kinase inhibitors AMG-18 and KIRA6 or the RNase inhibitor 4μ8c decreased IL-1β and Casp-1 secretion (Figure 5B).

To further analyze the role of IRE1 in myeloid cells during LCWE-induced KD vasculitis, we generated mice with myeloid cell–specific IRE1 deficiency by crossing mice harboring a Cre recombinase under the control of the endogenous *Lyz2* promoter (LysM^Cre^) with *Ern1^fl/fl^* mice (40). Western blot analysis confirmed *Ern1*-specific deletion in BMDMs from the resulting *LysM^Cre+^* *Ern1*^Δ*/*Δ^ mice, as IRE1 protein could not be detected in *LysM^Cre+^* *Ern1*^Δ*/*Δ^ BMDM cell lysates (Figure 5C). LCWE stimulation increased mRNA expression of the spliced form of *Xbp1* (*sXbp1*), a marker of UPR induction, in *Ern1^fl/fl^* BMDMs, but not in BMDMs generated from *LysM^Cre+^* *Ern1*^Δ*/*Δ^ mice (Figure 5D). Furthermore, LCWE stimulation increased mRNA levels of *Il1b*, *Il6*, and *Tnfa* in BMDMs derived from *Ern1^fl/fl^* mice, and IRE1 deletion in BMDMs resulted in the attenuation of LCWE-induced *Il1b* and *Il6* mRNA expression (Figure 5D). LCWE stimulation also induced p-IRE1 only in *Ern1^fl/fl^* BMDMs (Figure 5C). LCWE-induced inflammasome activation, measured by mature IL-1β and cleaved caspase-1 secretion, was also decreased in BMDMs isolated from *LysM^Cre+^ Ern1*^Δ*/*Δ^ mice (Figure 5C). Overall, our data indicate that genetic ablation of IRE1 signaling in macrophages or pharmacological inhibition of IRE1 reduces LCWE-induced inflammatory cytokine mRNA expression and NLRP3 inflammasome activation.

### Myeloid cell–specific IRE1 deficiency reduces the development of cardiovascular lesions during LCWE-induced KD vasculitis.

**Figure 6:**
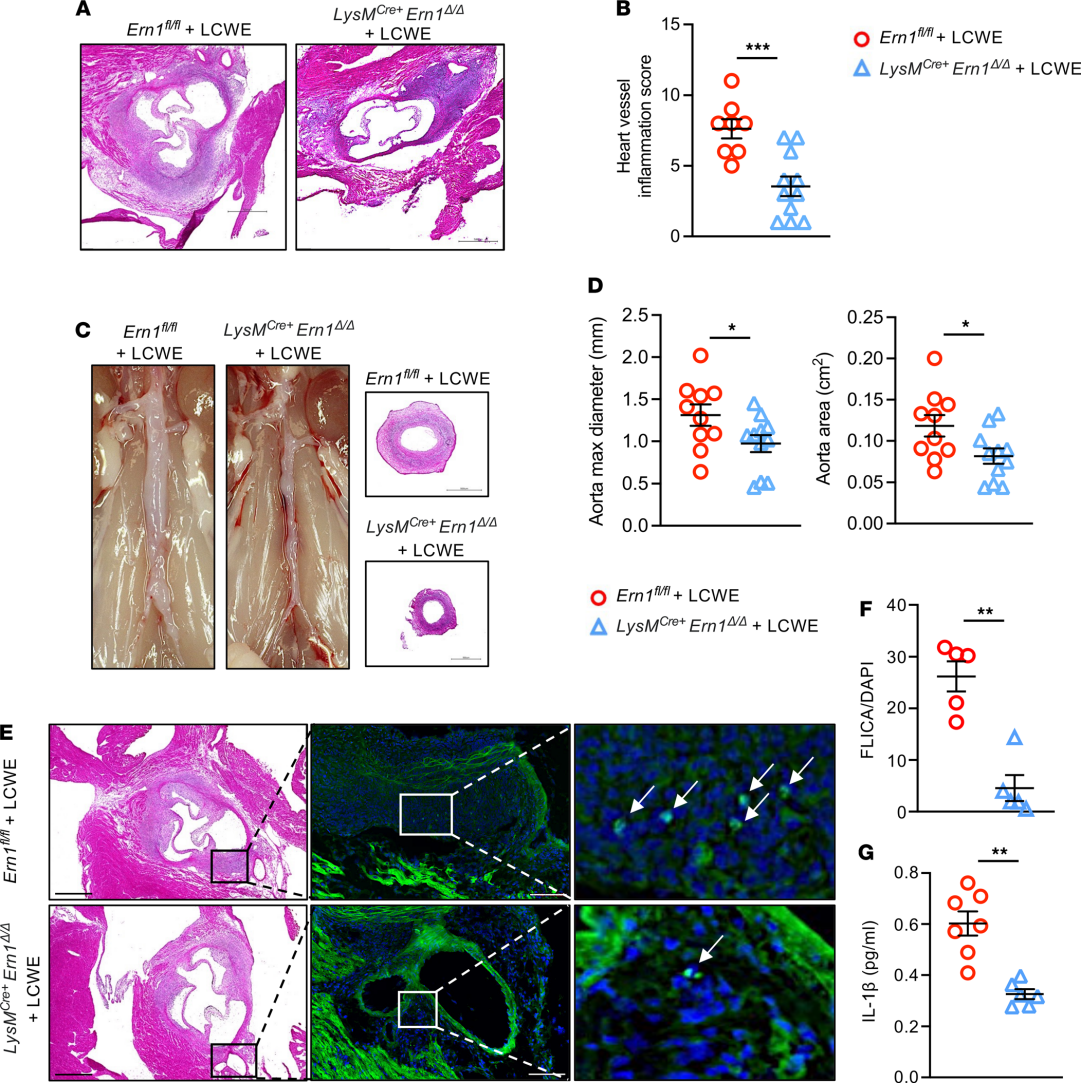
Myeloid cell–specific IRE1 deficiency reduces the development of LCWE-induced KD cardiovascular lesions. (**A** and **B**) Representative H&E staining of heart sections (**A**) and heart vessel inflammation score (**B**) of LCWE-injected *Ern1^fl/fl^* (*n* = 8) and LCWE-injected *LysM^Cre+^* *Ern1*^Δ*/*Δ^ (*n* = 11) mice at 2 weeks after LCWE injection. (**C** and **D**) Representative pictures of the abdominal aorta area, H&E staining of abdominal aorta cross section (**C**), and maximal abdominal aorta diameter and aorta area measurements (**D**) of LCWE-injected *Ern1^fl/fl^* (*n* = 10) and LCWE-injected *LysM^Cre+^* *Ern1*^Δ*/*Δ^ (*n* = 11) mice at 2 weeks after LCWE injection. Scale bars, 500 μm. (**E** and **F**) H&E staining, FLICA staining (**E**), and quantification (**F**) in heart tissues of LCWE-injected *Ern1^fl/fl^* (*n* = 5) and LCWE-injected *LysM^Cre+^* *Ern1*^Δ*/*Δ^ (*n* = 5) mice at 2 weeks after LCWE injection. White arrows indicate FLICA^+^ cells. Scale bars H&E staining: 500 μm; scale bars FLICA: 100 μm. (**G**) Serum IL-1β levels in LCWE-injected *Ern1^fl/fl^* (*n* = 7) and LCWE-injected *LysM^Cre+^* *Ern1*^Δ*/*Δ^ (*n* = 6) mice at 2 weeks after LCWE injection. *P < 0.05, **P < 0.01, ***P < 0.001 by Student’s *t* test (**B** and **D**) and Mann-Whitney test (**F** and **G**). FLICA; fluorescent labeled inhibitor of caspases assay.

To further determine the role of IRE1 in myeloid cells during LCWE-induced KD vasculitis, control *Ern1^fl/fl^* mice and *LysM^Cre+^ Ern1*^Δ*/*Δ^ mice were injected with LCWE. Compared with control mice, mice with IRE1 deficiency in myeloid cells showed reduced heart vessel inflammation following LCWE injection (Figure 6, A and B). The development of abdominal aorta aneurysms, measured by maximal abdominal aorta diameter and area, was also significantly decreased in myeloid-specific IRE1-deficient mice (Figure 6, C and D). Additionally, Casp-1 activity, measured in cardiovascular lesions using the fluorescent-labeled inhibitor of caspases assay (FLICA), as well as serum IL-1β levels, were reduced in LCWE-injected *LysM^Cre+^* *Ern1*^Δ*/*Δ^ mice compared with LCWE-injected *Ern1^fl/fl^* littermate controls (Figure 6, E–G).

### Pharmacological inhibition of IRE1 RNase activity reduces the development of cardiovascular lesions during LCWE-induced KD vasculitis.

**Figure 7:**
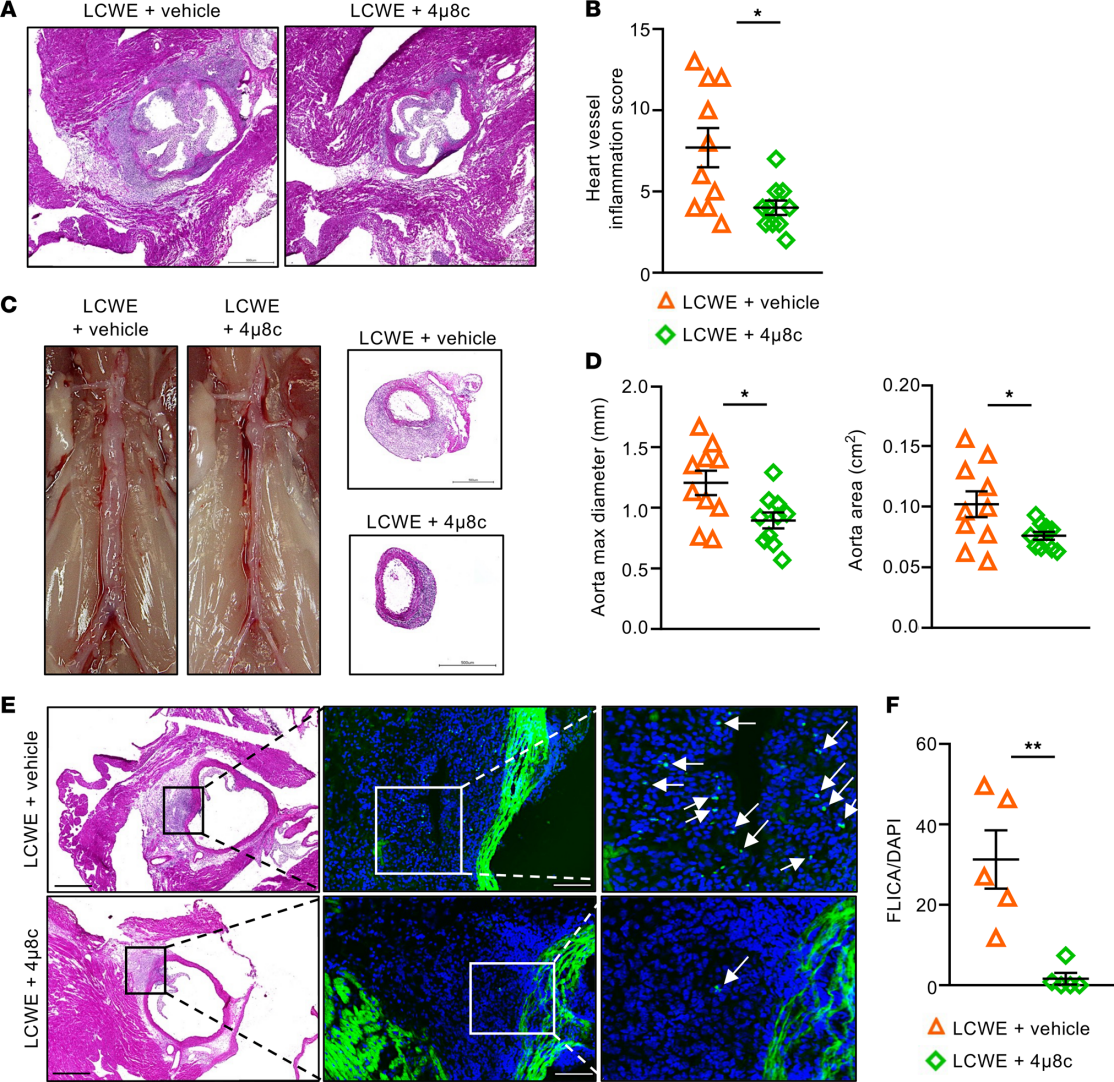
Pharmacological IRE1 inhibition with the small-molecule inhibitor 4μ8c reduces cardiovascular lesion formation in the LCWE murine model of KD vasculitis. (**A** and **B**) Representative H&E staining of heart sections (**A**) and heart vessel inflammation score (**B**) of LCWE-injected WT mice treated with either vehicle or 4μ8c, 1 week after LCWE injection (*n* = 10/group). (**C** and **D**) Representative pictures of the abdominal aorta area, H&E staining of abdominal aorta cross section (**C**), and maximal abdominal aorta diameter and aorta area measurements (**D**) of LCWE-injected WT mice treated with vehicle or 4μ8c, 1 week after LCWE injection (*n* = 10/group). Scale bars, 500 μm. (**E** and **F**) H&E staining, FLICA staining (**E**), and quantification (**F**) in heart tissues of LCWE-injected WT mice treated with either vehicle or 4μ8c, 1 week postinjection (*n* = 5/group). White arrows indicate FLICA^+^ cells. Scale bars H&E staining: 500 μm; scale bars FLICA: 100 μm. *P < 0.05 and **P < 0.01 by Student’s *t* test with Welch’s correction (**B** and **D**) and Mann-Whitney test (**F**).

We next determined if IRE1 could be therapeutically targeted to prevent or decrease the severity of LCWE-induced KD cardiovascular lesions. WT mice were treated daily with either vehicle or 4μ8c, an IRE1 RNase inhibitor that reduces the IL-1β signaling pathway in atherosclerosis (27), beginning 2 days prior to LCWE injection. Compared with LCWE-injected mice that received vehicle injection, pharmacological inhibition of IRE1 with 4μ8c resulted in reduced heart inflammation (Figure 7, A and B) as well as decreased development of abdominal aorta dilations (Figure 7, C and D). Casp-1 activity, measured by FLICA staining, was also significantly reduced in LCWE-injected mice that received the 4μ8c compared with vehicle-treated controls (Figure 7, E and F). Altogether, these findings are consistent with our in vitro observations and indicate that genetic or pharmacological targeting of IRE1 is protective against LCWE-induced KD vasculitis.

## Discussion

While there is strong evidence supporting the crucial role of NLRP3 inflammasome activation and IL-1β secretion in cardiovascular lesion development during human KD and in murine models of KD vasculitis (7, 10, 41, 42), and ER stress is an established inducer of NLRP3 inflammasome activity (33, 43), the role of ER stress during KD vasculitis has not previously been investigated to our knowledge. Here, we asked whether ER stress plays a causal role in KD vasculitis. Based on publicly available human patient transcriptomics data sets, bulk RNA-Seq, and scRNA-Seq from murine KD vasculitis, we show that KD development is associated with increased expression of ER stress signature genes, including IRE1/XBP1 target genes. Furthermore, protein expression of ERp57, an ER stress marker, was markedly upregulated in heart tissue of LCWE-injected mice developing vascular inflammation. This is consistent with other studies showing a strong association between ER stress and cardiovascular diseases, such as ischemic heart disease, hypertension, and atherosclerosis (44, 45).

We found that p-IRE1 was upregulated in abdominal aorta tissue of LCWE-treated mice. Activation of IRE1 by phosphorylation under conditions of ER stress leads to NLRP3 activation and consequent IL-1β secretion, a mechanism associated with inflammatory processes in cardiovascular tissue (27, 46). In conjunction with these published findings, our data suggest that IRE1 activation contributes to cardiovascular lesion formation in the LCWE-induced murine model of KD vasculitis. Indeed, BMDMs from WT mice showed dose-dependent increases in p-IRE1 and ERp57 protein expression after LCWE stimulation. This is notable because myeloid cells are the main IL-1β producers in LCWE-induced cardiovascular lesions (9, 10, 39), and IL-1β transcripts are mainly expressed by circulating peripheral blood monocytes in patients with acute KD (47). To further assess the role of the IRE1 pathway in myeloid cells in the LCWE murine model of KD vasculitis, we generated *LysΜ^Cre+^* *Ern1*^Δ*/*Δ^ mice, in which myeloid cells are IRE1 deficient. After stimulation with LCWE, BMDMs from *LysΜ^Cre+^* *Ern1*^Δ*/*Δ^ mice showed reduced mRNA levels of *Il1b* compared with BMDMs generated from *Ern1^fl/fl^* controls, indicating that inhibition of the IRE1 pathway may lead to reduced NLRP3 activation, consistent with previous findings in other types of cardiovascular diseases (27, 46). Protein levels of mature IL-1β and cleaved caspase-1 were also downregulated in BMDMs from *LysΜ^Cre+^* *Ern1*^Δ*/*Δ^ mice compared with those from *Ern1^fl/fl^* mice. Consistent with these observations in macrophages, *LysΜ^Cre+^* *Ern1*^Δ*/*Δ^ mice displayed reduced heart inflammation and abdominal aorta aneurysm development compared with *Ern1^fl/fl^* mice. In these myeloid cell–specific IRE1-deficient mice, systemic IL-1β levels and local Casp-1 activity in heart tissue were also reduced. These findings confirm that activation of IRE1 by ER stress plays an important role in the LCWE murine model of KD vasculitis.

We asked whether targeting the IRE1 pathway by a small-molecule inhibitor could also attenuate cardiovascular lesion formation in LCWE-dependent KD vasculitis. Treatment with an inhibitor of IRE1 RNase activity, 4μ8C, reduced heart inflammation and abdominal aorta aneurysm formation in LCWE-injected mice. Consistent with previous findings in an atherosclerosis model (27), 4μ8C treatment led to a reduction of Casp-1 activity in the hearts of LCWE-injected mice, reflecting reduced NLRP3 inflammasome activation. Our combined in vitro and in vivo data strongly suggest that IRE1 inhibition attenuates cardiovascular lesion formation in the murine KD vasculitis by diminishing NLRP3 activation.

Mitochondria and ER are in close physical and functional contact. They exchange information and molecules important for many cellular processes, including synthesis of phospholipids, formation of autophagosomes, and mitochondrial fission (48–53). This interface is also an important platform for NLRP3 inflammasome assembly (54). Stress in either organelle is effectively transferred to the other, leading to increased ROS production from both and formation of damage-associated molecular patterns, required for robust activation of the NLRP3 inflammasome (55). We have previously reported that LCWE-induced KD vasculitis is associated with impaired autophagy/mitophagy and increased ROS production in inflamed vascular tissues, which promotes NLRP3 inflammasome activation and the development of cardiovascular lesions in LCWE-injected mice (56). Our observations that IRE1 plays an upstream role in NLRP3 signaling are also in accordance with prior findings regarding the association of ER stress with cardiovascular disease (27, 44).

Studies report that circulating levels of IL-1β are increased during the acute phase of KD and reduced following IVIG treatment (13, 14). Transcriptomics analysis of whole blood from patients with KD that is responsive or resistant to IVIG treatment also indicates increased abundance of multiple genes belonging to the IL-1 pathway in IVIG-resistant patients (15). These observations led to the development of clinical trials testing anakinra, an IL-1 receptor antagonist, for the treatment of IVIG-resistant patients (21, 57, 58). Here, we show that the development of cardiovascular lesions in a murine model of KD vasculitis is associated with increased ER stress. We also demonstrate that pharmacological modulation of the IRE1 pathway hampers NLRP3 inflammasome activation and IL-1β secretion, resulting in decreased development of cardiovascular lesions. Our study demonstrates not only that IRE1 signaling in myeloid cells contributes to LCWE-induced vasculitis development but also that the inhibition of IRE1 RNase activity by a highly specific small molecule can counteract cardiovascular lesions in this model, highlighting IRE1 as a therapeutic target in KD. Whether IVIG-resistant KD patients would benefit from targeting of organelle stress responses or whether treatment with IRE1 inhibitors could prevent long-term cardiovascular sequelae warrant deeper investigation. The ongoing efforts to discover next-generation IRE1 kinase and RNase inhibitors make these exciting times to further explore the potential of IRE1 modulation in KD vasculitis.

## Methods

### Mice.

C57BL/6J WT mice and *LysM^Cre^* (004781) mice were purchased at the Jackson Laboratory. IRE1–conditional knockout (*Ern1^fl/fl^*) mice were a gift from Kanazawa Medical University and characterized previously (40). *Ern1^fl/fl^* mice were crossed with *LysM^Cre^* mice to obtain *LysM^Cre+^* *Ern1*^Δ*/*Δ^ mice, which had a myeloid cell–specific IRE1 gene deletion, as previously described (59). As LCWE injection induces a stronger vasculitis in male mice than female mice, only male mice were used in this study (9, 11). Mice were housed under specific pathogen–free conditions and used according to the guidelines of the Cedars-Sinai Medical Center IACUC.

### LCWE-induced KD vasculitis murine model.

*Lactobacillus casei* (ATCC 11578) cell wall extract (LCWE) was prepared in our laboratory as previously described (8). Male mice at 5 weeks old were injected with a single dose of 500 μg of LCWE or PBS i.p. Depending of the experimental design, mice were euthanized 1 or 2 weeks after injection. Blood, heart, and abdominal aorta tissues were collected and tissues embedded in Optimal Cutting Temperature (Tissue-Tek O.C.T. Compound, Sakura Finetek, 4583) for further analysis, as previously described (8–11). Abdominal aortas were photographed prior to embedding, and the infrarenal part located between the left renal artery and common iliac branches was used to quantify the area and maximal diameter (measured at 5 areas) with ImageJ (NIH), as previously described (9–11). Frozen heart and abdominal aorta tissues were cut into serial cryosections (7 μm) and stained with H&E. Histopathological scoring of the coronary arteritis and aortic root vasculitis was performed on H&E-stained heart tissue sections using a blinded protocol as previously published (8, 10).

### Immunofluorescence.

To assess Casp-1 and NLRP3 inflammasome activation, heart tissue cryosections were stained with the FAM-FLICA Caspase-1 Assay kit (ImmunoChemistry Technologies) according to the manufacturer’s protocol. Signal quantification was performed by calculating the corrected total cell fluorescence.

### In vivo pharmacological IRE1 inhibition.

Cremophor (MilliporeSigma, 238470) was heated at 37°C to achieve liquidity and then mixed with sterile saline to obtain a 20% Cremophor solution. IRE1 Inhibitor III, 4μ8C (MilliporeSigma, CAS 14003-96-4), was resuspended in the 20% Cremophor solution and injected i.p. into the mice at a concentration of 10 mg/kg/d. Mice were injected daily, beginning 2 days before LCWE injection until day 7 after LCWE and tissue harvest. The drug was prepared fresh every day.

### Immunoprecipitation.

Abdominal aortas from vehicle or LCWE-injected C57BL/6J mice were lysed with phospholysis buffer (PLB: 50 mM HEPES pH: 7.9, 100 mM NaCl, 10 mM EDTA, 10 mM NaF, 4 mM Na_4_P_2_O_7_, 1% Triton, 2 mM Na_3_VO_4_, 1 mM PMSF, 1× phosphatase inhibitor cocktail 3, and 1 × [10 μM/mL] protease inhibitor cocktail) by sonication and immunoprecipitated with either anti–IgG-R– or anti-IRE1–conjugated magnetic beads (Protein G Magnetic Beads, BioRad 1614023). Beads were washed with PLB and boiled upon adding 5× SDS loading buffer for 5 minutes at 95°C. Proteins were then separated by electrophoresis in SDS-PAGE gels for analysis. Immunoprecipitated lysates from either DMSO-treated or thapsigargin-treated (Santa Cruz Biotechnology, sc-24017A) HEK293T cells (ATCC) were used as controls.

### BMDM isolation and culture.

Bone marrow isolation and culture were done as previously described (59). Briefly, bone marrow was isolated from tibia and femur bones using a 1 mL syringe with 22G needle by flushing RPMI containing 1% penicillin/streptomycin (P/S). After homogenization, cell suspension was filtered through a 70 μm cell strainer, and cells were cultured in RPMI (Corning, 10-041-CV) containing 10% fetal bovine serum, 1% P/S, and 20% L929 cell–conditioned (ATCC) medium and grown on non–cell culture–grade, sterile Petri dishes for 5 days. BMDMs were pretreated with IRE1 kinase domain inhibitors AMG-18 (3 μM, Tocris, 6166), KIRA6 (15 μM, Cayman Chemical, 19151), and RNase domain inhibitor 4μ8c (100 μM, MilliporeSigma, 412512) for 1 hour and then treated with LCWE (75 μg/mL) for 14 hours along with the inhibitors. TM (2.5 and 5 μg/mL; Santa Cruz Biotechnology, 3506) was used as an ER stress inducer positive control.

### Western blot.

Whole-cell lysates were prepared in a PLB, as described previously (59). Lysates were cleared by centrifugation at 8000*g* for 10 minutes at 4°C followed by the addition of 5× SDS-loading dye and heated at 95°C for 5 minutes before loading on SDS-PAGE gels. Samples were transferred to PVDF membranes. Blocking and antibody incubation of the membranes were carried out in Tris-buffered saline buffer prepared with 0.1% Tween 20 (*v*/*v*) and 5% (*w*/*v*) dry milk or BSA. The membranes were incubated with the following antibodies: anti–phospho-S724 IRE1 (Abcam, 124945), IRE1 antibody (Cell Signaling Technology, 3294), anti–IL-1β antibody (Abcam, 9722), Anti–pro Casp-1 (Abcam, 179515), ERp57 (Santa Cruz Biotechnology, sc-23886), and β-Actin–HRP (Santa Cruz Biotechnology, 47778). Membranes were developed in ECL prime reagent (Amersham), and images were captured with ChemiDoc (Bio-Rad).

### RNA isolation and qRT-PCR.

TRIzol reagent (Thermo Fisher Scientific) was used to isolate total RNA from cells. The RNA was reverse-transcribed to cDNA using Revert-Aid First Strand cDNA synthesis kit (Thermo Fisher Scientific, K1691) according to manufacturer’s protocol. Power-Up-SYBR green was used for the qRT-PCR reaction on a Rotor Gene qRT-PCR machine (QIAGEN). The following PCR primers were used for mRNA expression analysis: *sXbp1*: F′ TGAGAACCAGGAGTTAAGAACACGC, R′ CCTGCACCTGCTGCGGAC; *Il1b*: F′ CAACCAACAAGTGATATTCTCCAT, R′ GATCCACACTCTCCAGCTGCA; *Tnfa*: F′ CATCTTCTCAAAATTCGAGTGACAA, R′ TGGGAGTAGACAAGGTACAACCC; *Il6*: F′ GAGGATACCACTCCCAACAGACC, R′ AAGTGCATCATCGTTGTTCATACA; and *Gapdh*: F′ GTGAAGGTCGGTGTGAACG, R′ GGTCGTTGATGGCAACAATCTC.

### ELISA.

IL-1β was measured in serum with the V-PLEX Mouse IL-1β Kit (Meso Scale Diagnostics, K152QPD-1) per manufacturer’s instructions. The samples were read and analyzed by MSD QuickPlex SQ120 instrumentation and Workbench 4.0 Software (Meso Scale Diagnostics).

### Abdominal aorta scRNA-Seq.

Expression of ER stress–related genes was assessed on a publicly available scRNA-Seq data set generated from the abdominal aortas of PBS-injected (pool of *n* = 9) and LCWE-injected (pool of *n* = 7) mice (National Center for Biotechnology Information’s Gene Expression Omnibus GSE178765), as previously published (39). After quality control and exclusion of cells with fewer than 1000 expressed genes and exclusion of doublets, UMAPs were performed using the R package *umap* v0.2.2.0 (60), and cell clustering and clusters annotations were done with Seurat V3 and SingleR, as previously described (10).

### Analysis of murine abdominal aorta gene expression data set.

ER stress signature genes’ expression was also analyzed in a murine RNA-Seq data set generated from abdominal aortas of PBS-injected (*n* = 5) and LCWE-injected (*n* = 5) mice (GSE141072) (11). Normalization and analysis of gene expression data were performed in R using edgeR and limma-voom, as previously published (11). ER stress signature genes were considered DEGs with an adjusted *P* < 0.05 and FC ≥ 1.5. Heatmaps showing the expression relative to the mean of selected genes were generated in R with the “ComplexHeatmap” package.

### Analysis of human gene expression data sets.

Publicly available gene expression data sets GSE68004 (35), GSE73461 (36), and GSE63881 (37) were obtained from National Center for Biotechnology Information’s Gene Expression Omnibus (GEO; https://www.ncbi.nlm.nih.gov/geo/). Transcriptomic data analysis was run by GEO2R software as part of the GEO database, and summary statistics were generated with limma topTable function. The following ER stress signature genes were selected*:* *ATF4*, *ATF6*, *CEBPB*, *DDIT3*, *DNAJB11*, *DNAJC3*, *EDEM1*, *EDEM2*, *EIF2A*, *EIF2AK2*, *EIF2AK3*, *ERN1*, *ERP29*, *ERP44*, *HSPA5*, *OS9*, *PDIA3*, *PDIA4*, *PDIA6*, *RTN3*, *S100P*, *SEL1L*, *SOD2*, *SSR1*, *TRIB1*, *TRIB3*, *WFS1*, *WIPI1*, *XBP1*. Their expression was assessed in whole blood of patients with acute KD and HCs (*n* = 37 HCs and *n* = 76 acute KD patients for GSE68004; ref. 35; *n* = 55 HCs and *n* = 78 acute KD patients for GSE73461; ref. 36). DEGs (Benjamini-Hochberg–adjusted *P* < 0.05 and FC > 1.5) from the ER gene signature were identified in these 2 data sets (GSE68004 and GSE73461), and their expression was subsequently analyzed in the whole blood of acute KD patients (*n* = 146) and IVIG-treated convalescent KD patients (*n* = 145) (adjusted *P* < 0.05) (GSE63881) (37).

### Statistics.

Data were analyzed with Prism software (GraphPad) and are presented as mean ± SEM. Normality of data was assessed with the Shapiro-Wilk (Figure 5D) or D’Agostino-Pearson normality tests with α = 0.05. For 2-group comparisons of normally distributed data, Student’s unpaired 2-tailed *t* tests, with Welch’s correction when indicated, were used. For nonparametric data, the Mann-Whitney *U* test was used. For multiple-comparison testing, significance was evaluated by 2-way ANOVA with Tukey’s post hoc test. *P* < 0.05 was considered significant.

### Study approval.

All animal studies in this manuscript were approved by the IACUC of Cedars-Sinai Medical Center and were performed in accordance with the *Guide for the Care and Use of Laboratory Animals* (National Academies Press, 2011).

## Author contributions

EE and MA conceptualized the study. SMI, ADY, SMH, ABO, ACG, BK, and RAP performed experiments. MNR, EE, and MA supervised experiments. Data analysis was performed by SMI, ADY, SMH, BK, RAP, MCF, and MNR. Data discussion was contributed by SMI, ADY, SMH, BK, RAP, MNR, EE, and MA. TI provided critical reagents and contributed to the conception of this study. Manuscript writing was contributed by SMI, ADY, RAP, MNR, EE, and MA. The order of equally contributing authors was decided by seniority and funding of the study.

## Figures and Tables

**Table 1:**
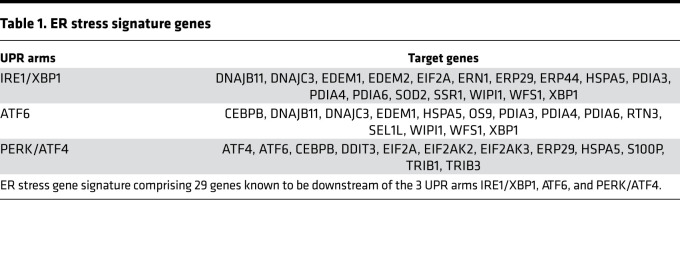
ER stress signature genes

## Version 1 02/15/2022

In-Press Preview

## Version 2 03/22/2022

Electronic publication

## References

[1] Soni PR (2020). A comprehensive update on Kawasaki Disease vasculitis and myocarditis. Curr Rheumatol Rep.

[2] Newburger JW (2016). Kawasaki disease. J Am Coll Cardiol.

[3] McCrindle BW (2017). Diagnosis, treatment, and long-term management of Kawasaki disease: a scientific statement for health professionals from the American Heart Association. Circulation.

[4] Burns JC (2009). Kawasaki disease update. Indian J Pediatr.

[5] Skochko SM (2018). Kawasaki disease outcomes and response to therapy in a multiethnic community: a 10-year experience. J Pediatr.

[6] Noval Rivas M (2017). CD8+ T cells contribute to the development of coronary arteritis in the lactobacillus casei cell wall extract-induced murine model of Kawasaki disease. Arthritis Rheumatol.

[7] Noval Rivas M, Arditi M (2020). Kawasaki disease: pathophysiology and insights from mouse models. Nat Rev Rheumatol.

[8] Lee Y (2012). Interleukin-1β is crucial for the induction of coronary artery inflammation in a mouse model of Kawasaki disease. Circulation.

[9] Wakita D (2016). Role of interleukin-1 signaling in a mouse model of Kawasaki disease-associated abdominal aortic aneurysm. Arterioscler Thromb Vasc Biol.

[10] Porritt RA (2021). NLRP3 inflammasome mediates immune-stromal interactions in vasculitis. Circ Res.

[11] Porritt RA (2020). Interleukin-1 beta-mediated sex differences in Kawasaki disease vasculitis development and response to treatment. Arterioscler Thromb Vasc Biol.

[12] Maury CP (1988). Circulating interleukin-1 beta in patients with Kawasaki disease. N Engl J Med.

[13] Leung DY (1989). Endothelial cell activation and high interleukin-1 secretion in the pathogenesis of acute Kawasaki disease. Lancet.

[14] Alphonse MP (2016). Inositol-triphosphate 3-kinase C mediates inflammasome activation and treatment response in Kawasaki disease. J Immunol.

[15] Fury W (2010). Transcript abundance patterns in Kawasaki disease patients with intravenous immunoglobulin resistance. Hum Immunol.

[16] Weng KP (2010). IL-1B polymorphism in association with initial intravenous immunoglobulin treatment failure in Taiwanese children with Kawasaki disease. Circ J.

[17] Cohen S (2012). A child with severe relapsing Kawasaki disease rescued by IL-1 receptor blockade and extracorporeal membrane oxygenation. Ann Rheum Dis.

[18] Shafferman A (2014). High dose anakinra for treatment of severe neonatal Kawasaki disease: a case report. Pediatr Rheumatol Online J.

[19] Guillaume MP (2018). Usefulness and safety of anakinra in refractory Kawasaki disease complicated by coronary artery aneurysm. Cardiol Young.

[20] Blonz G (2020). Severe late-onset Kawasaki disease successfully treated with anakinra. J Clin Rheumatol.

[21] Kone-Paut I (2018). The use of interleukin 1 receptor antagonist (anakinra) in Kawasaki disease: a retrospective cases series. Autoimmun Rev.

[22] Hetz C (2020). Mechanisms, regulation and functions of the unfolded protein response. Nat Rev Mol Cell Biol.

[23] Walter P, Ron D (2011). The unfolded protein response: from stress pathway to homeostatic regulation. Science.

[24] Hotamisligil GS (2010). Endoplasmic reticulum stress and the inflammatory basis of metabolic disease. Cell.

[25] Basseri S, Austin RC (2012). Endoplasmic reticulum stress and lipid metabolism: mechanisms and therapeutic potential. Biochem Res Int.

[26] Onat UI (2019). Intercepting the lipid-induced integrated stress response reduces atherosclerosis. J Am Coll Cardiol.

[27] Tufanli O (2017). Targeting IRE1 with small molecules counteracts progression of atherosclerosis. Proc Natl Acad Sci U S A.

[28] Calfon M (2002). IRE1 couples endoplasmic reticulum load to secretory capacity by processing the XBP-1 mRNA. Nature.

[29] Yoshida H (2001). XBP1 mRNA is induced by ATF6 and spliced by IRE1 in response to ER stress to produce a highly active transcription factor. Cell.

[30] Robblee MM (2016). Saturated fatty acids engage an IRE1α-dependent pathway to activate the NLRP3 inflammasome in myeloid cells. Cell Rep.

[31] Chen X (2019). ER stress activates the NLRP3 inflammasome: a novel mechanism of atherosclerosis. Oxid Med Cell Longev.

[32] Lebeaupin C (2015). ER stress induces NLRP3 inflammasome activation and hepatocyte death. Cell Death Dis.

[33] Bronner D (2015). Endoplasmic reticulum stress activates the inflammasome via NLRP3- and caspase-2-driven mitochondrial damage. Immunity.

[34] Rossol M (2012). Extracellular Ca2+ is a danger signal activating the NLRP3 inflammasome through G protein-coupled calcium sensing receptors. Nat Commun.

[35] Jaggi P (2018). Whole blood transcriptional profiles as a prognostic tool in complete and incomplete Kawasaki disease. PLoS One.

[36] Wright VJ (2018). Diagnosis of Kawasaki disease using a minimal whole-blood gene expression signature. JAMA Pediatr.

[37] Hoang LT (2014). Global gene expression profiling identifies new therapeutic targets in acute Kawasaki disease. Genome Med.

[38] Lerner AG (2012). IRE1α induces thioredoxin-interacting protein to activate the NLRP3 inflammasome and promote programmed cell death under irremediable ER stress. Cell Metab.

[39] Lee Y (2015). IL-1 Signaling is critically required in stromal cells in Kawasaki disease vasculitis mouse model: role of both IL-1α and IL-1β. Arterioscler Thromb Vasc Biol.

[40] Iwawaki T (2009). Function of IRE1 alpha in the placenta is essential for placental development and embryonic viability. Proc Natl Acad Sci U S A.

[41] Burns JC (2017). Review: found in translation: international initiatives pursuing interleukin-1 blockade for treatment of acute Kawasaki disease. Arthritis Rheumatol.

[42] Anzai F (2019). Crucial role of NLRP3 inflammasome in a murine model of Kawasaki disease. J Mol Cell Cardiol.

[43] Menu P (2012). ER stress activates the NLRP3 inflammasome via an UPR-independent pathway. Cell Death Dis.

[44] Yang S (2020). Role of endoplasmic reticulum stress in atherosclerosis and its potential as a therapeutic target. Oxid Med Cell Longev.

[45] Ren J (2021). Endoplasmic reticulum stress and unfolded protein response in cardiovascular diseases. Nat Rev Cardiol.

[46] Hong J (2017). The role of endoplasmic reticulum stress in cardiovascular disease and exercise. Int J Vasc Med.

[47] Wang Z (2021). Single-cell RNA sequencing of peripheral blood mononuclear cells from acute Kawasaki disease patients. Nat Commun.

[48] Murley A, Nunnari J (2016). The emerging network of mitochondria-organelle contacts. Mol Cell.

[49] Marchi S (2014). The endoplasmic reticulum-mitochondria connection: one touch, multiple functions. Biochim Biophys Acta.

[50] Friedman JR (2011). ER tubules mark sites of mitochondrial division. Science.

[51] De Stefani D (2011). A forty-kilodalton protein of the inner membrane is the mitochondrial calcium uniporter. Nature.

[52] Baughman JM (2011). Integrative genomics identifies MCU as an essential component of the mitochondrial calcium uniporter. Nature.

[53] Stone SJ, Vance JE (2000). Phosphatidylserine synthase-1 and -2 are localized to mitochondria-associated membranes. J Biol Chem.

[54] Zhou R (2011). A role for mitochondria in NLRP3 inflammasome activation. Nature.

[55] Guo H (2015). Inflammasomes: mechanism of action, role in disease, and therapeutics. Nat Med.

[56] Marek-Iannucci S (2021). Autophagy-mitophagy induction attenuates cardiovascular inflammation in a murine model of Kawasaki disease vasculitis. JCI Insight.

[57] Tremoulet AH (2016). Rationale and study design for a phase I/IIa trial of anakinra in children with Kawasaki disease and early coronary artery abnormalities (the ANAKID trial). Contemp Clin Trials.

[58] Koné-Paut I (2021). Phase II open label study of anakinra in intravenous immunoglobulin-resistant Kawasaki disease. Arthritis Rheumatol.

[59] Hamid SM (2020). Inositol-requiring enzyme-1 regulates phosphoinositide signaling lipids and macrophage growth. EMBO Rep.

[60] Becht E Dimensionality reduction for visualizing single-cell data using UMAP. Nat Biotechnol.

